# Improved human visuomotor performance and pupil constriction after choline supplementation in a placebo-controlled double-blind study

**DOI:** 10.1038/srep13188

**Published:** 2015-08-14

**Authors:** Marnix Naber, Bernhard Hommel, Lorenza S. Colzato

**Affiliations:** 1 Leiden University, Cognitive Psychology, Wassenaarseweg 52, Leiden, 2333 AK, The Netherlands; 2 Leiden Institute for Brain and Cognition, Leiden University Medical Center, P.O. Box 9600, Leiden, 2300 RC, The Netherlands

## Abstract

Only few nutrients are known to enhance cognition. Here we explore whether visuomotor performance can be improved through the intake of the nutrient choline, an essential chemical compound in a vertebrate’s diet. Choline is abundant in for example eggs and shrimps and many animal studies suggest that it serves as a cognitive enhancer. As choline is important for the communication between motor neurons and the control of skeletal muscles, we assumed that choline supplementation may have positive effects on action coordination in humans. A group of twenty-eight individuals ingested two grams of choline bitartrate or a placebo in two separate sessions. Seventy minutes post ingestion, participants performed a visuomotor aiming task in which they had to rapidly hit the centers of targets. Results showed that participants hit targets more centrally after choline supplementation. Pupil size (a cognition-sensitive biomarker) also significantly decreased after choline intake and correlated positively with the hit distance to the targets and the number of target misses, and negatively with reaction times. These findings point to a choline-induced bias towards action precision in the trade-off between speed and accuracy. The changes in pupil size suggest that choline uptake alters cholinergic functions in the nervous system.

Humans need to consume a complex set of nutrients to be able to stay alive and live healthy on the long-term. However, food ingestion can also have short-term effects, which among other things provides the opportunity to support cognitive functioning through functional food. Chemical compounds such as caffeine and nicotine are known to alter the state of arousal and performance on a variety of tasks^1^,^2^,^3^,^4^. These effects can act quickly and are relatively evident because they provide the user a conscious feeling of increased general alertness. There also exist nutrients with more subtle and often unnoticed effects that target and enhance specific cognitive skills^5^,^6^,^7^. These nutrients include amino acids such as tryptophan and tyrosine which are precursors of the neurotransmitters serotonin and dopamine. Choline might be an additional nutrient with effects on cognition.

Animal studies suggest that choline is the precursor of acetylcholine^8^,^9^,^10^,^11^,^12^, a neurotransmitter crucial for communication between neurons in the nervous system^13^,^14^. Several studies show that choline ingestion enhances memory in rodents^15^,^16^,^17^,^18^,^19^,^20^. Effects of choline-containing compounds on human memory are mixed with no improvements in memory by lecithin and choline chloride^21^,^22^,^23^,^24^,^25^ but significant enhancements in a wide variety of memory and executive control functions, including working memory (e.g., n-back task), verbal memory and learning (e.g., recall a list of words), and maze pathway learning after CDP-choline (citicoline) and alpha-GPC (choline alfoscerate) administration in populations that have relatively low baseline memory performance^26^,^27^,^28^,^29^,^30^. Memory and executive control can thus be improved by specific set of chemical substances with a choline element in people with mild to moderate memory impairments. Unfortunately, the positive effect of choline on cognition in healthy human populations has remained unstudied. Further, motor coordination, a cognitive function that also relies on cholinergic brain networks^31^, has received much less attention than memory by dietary choline studies. A study by Knott and colleagues^32^ found that only participants with low baseline performance on a variety of tasks had faster button presses to visual changes after CDP-choline supplementation. It remains unknown, however, how choline affects the spatial component (accuracy) and reaction times of aimed movements.

More interestingly for our purposes, there is some evidence for a link between choline ingestion and alteration of action behavior in rats. For example, Toide has shown that an increase in choline release in the hippocampus and frontal cortex is related to an increase in locomotor activity in rats^33^. Day *et al*. showed that the anticholinergic drug scopolamine alters choline release in the striatum^34^, a brain area crucial for the coordination of fine bodily movements^35^. Another study by Nag and Berger-Sweeney^36^ indicated that postnatal dietary choline can attenuate deficits in motor coordination and locomotor activity in male rodents with Rett syndrome, a neurodevelopmental disorder in cholinergic brain systems associated with impairments in motor functions^31^. Furthermore, Pacelli and colleagues demonstrated that dietary choline deprivation for multiple days causes impairments in motor coordination and motor learning in rats^37^. These studies suggest that the element choline plays a key role in motor functions in rodents. However, no studies have systematically looked into the effects of choline supplementation on motor coordination in healthy humans. The present study aimed to address this shortcoming by testing, in a placebo-controlled, double-blind study, whether humans can improve their visuomotor performance after the ingestion of choline. We hypothesized that choline supplementation results in improved coordinated movements of the arm. In addition to muscle contractions and relaxations through the somatic nervous system^14^, cholinergic functions are also the primary driver of pupil constrictions in the peripheral nervous system^38^. We considered that this connection, if it could be experimentally validated, provides the opportunity to use pupil size as a biomarker to monitor the efficiency of motor-related cognitive skills online. Accordingly, we expected that choline ingestion would also lead to (more) constricted pupils.

## Methods

### Participants

Thirty human individuals were invited to participate in the experiment. Behavioral measures were not acquired for the first participant due to technical start-up problems and another participant did not show up for the second session. The remaining 28 participants (age M = 19.50, SD = 2.05; 24 females; body mass index M = 22.50, SD = 2.69) had normal or corrected-to-normal vision, had no cardiac, hepatic, renal, neurological or psychiatric disorders, personal or family history of depression, migraine, and no medication or drug use. Female participants that used contraception were only tested outside their menstrual period to minimize confounds of hormonal differences (for details, see^39^). Participants were naïve to the purpose of the experiment and were told that they had to drink orange juice to investigate the effects of vitamin C on behavior. As such, treatments were deceptive because participants were not aware of the choline administration. To prevent a potential overdose of choline we measured blood pressure at several moments during the experiment (see below). The experiments conformed to the ethical principles of the Declaration of Helsinki and were approved by the local ethical committee (Leiden University, Institute for Psychological Research). All participants were right-handed students and received study credit for participation. Furthermore, all gave informed written consent at the start of the first session of the experiment and participants were debriefed after the second session.

### Apparatus and material

Depending on the session, participants were given 400 ml orange juice including 2 g dissolved choline bitartrate or a microcrystalline cellulose placebo, both consisting of a fine-grained, white powder that did not change the viscosity of the drink. Choline bitartrate contains 41.1% choline by molecular weight, and 2 gram of choline bitartrate administration provides 800 mg of choline action, similar to the ingestion of approximately 5 hard-boiled eggs or 250 g beef liver. The given amounts were well below the established 3.5 g recommended upper intake level for adults (Food and nutrition board of the US institute of Medicine). Choline uptake peaks approximately thirty minutes after ingestion^12^, and acetylcholine levels in brains significantly raise after approximately forty minutes^12^ and remain high for at least ninety minutes^11^. In addition, choline bitartrate and lecithin (phosphatidylcholine) increase choline plasma levels in humans within one hour after ingestion^40^,^41^,^42^,^43^.

Stimuli were generated on an Asus Vivobook laptop computer with Windows 8 operating system (Microsoft) and MatLab (Mathworks). The computers desktop was extended to a presentation monitor that displayed 1280 by 1024 pixels at a 60-Hz refresh rate. Screen size was 30 cm in width and 23 cm in height (31 by 25 visual degrees), and the participant’s viewing distance to the screen was fixed with a chin and forehead rest at approximately half a meter. Heart rate (HR) and systolic and diastolic blood pressure (SBP and DPB) was measured from the non-dominant arm with an OSZ3 automatic digital electronic wrist blood pressure monitor (Welch Allyn). Choline supplementation did not significantly affect these measurements as compared to placebo (*p*’s > 0.05). Images (640 by 480 pixels) of the pupil were recorded with a tripod mounted RGB Flea3 USB3 camera (Point Grey, Richmond, BC, Canada) with a Tokina AT-X 90 mm macro-lens at 60 Hz while participant’s fixated a small dot at the center of a screen with a grey background.

### Stimuli and procedure

**Figure 1:**
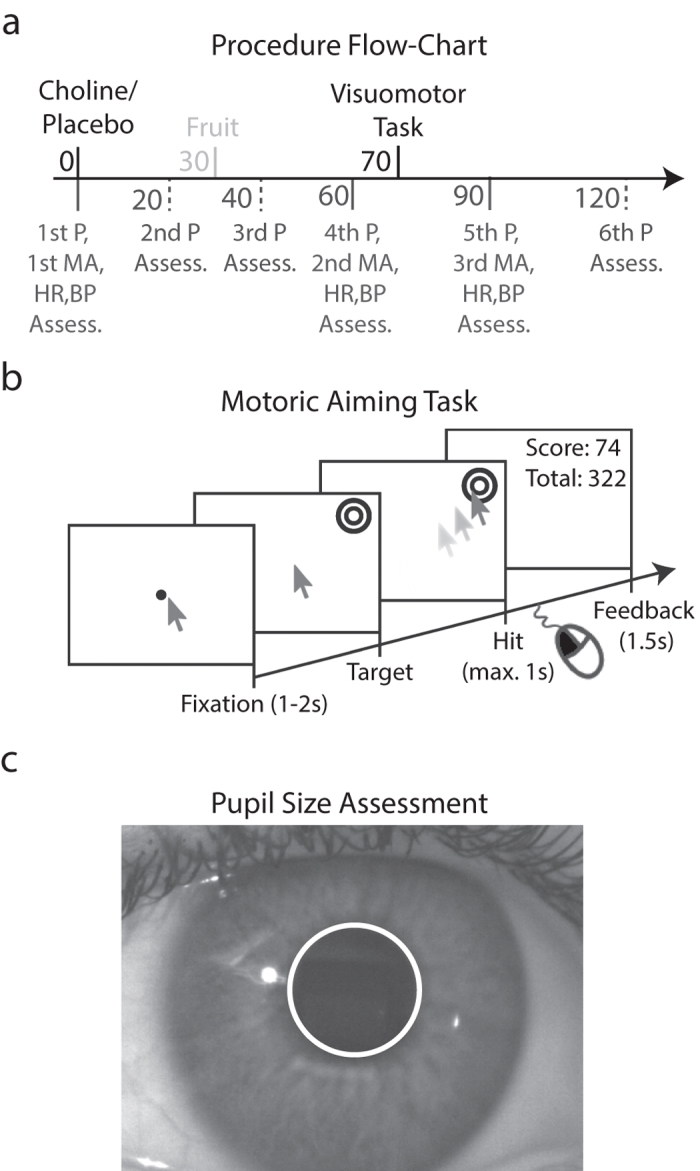
Methods. (**a**) Participants were given a 2 g choline bitartrate or placebo supplement and assessed for their mood and arousal (MA), heart rate (HR), and blood pressure (BP) after arrival at the lab, and right before and after the visuomotor task. Pupil size (P) was also measured at a 20 minute interval before the task and 30 minute interval after the task. Participants were allowed to consume one piece of fruit 30 minutes after supplementation and the visuomotor task was performed 40 minutes later. (**b**) During the task, participants had to move a mouse cursor and hit the center of a target as fast as possible (within one second) to accumulate scores for their accuracy and reaction time across trials. Scores were based on the hit distance to the target’s center and reaction times. (**c**) Pupil size was extracted from recorded images by determining the median radius of the pupil’s border (see white circle) from the center.

We tested healthy students on a visuomotor task, while recording their pupil size, approximately 70 minutes after the administration of 2 gram of choline bitartrate or a placebo substance (for a flow-chart, see Fig. 1a). To control for choline uptake unaffected by other supplements, participants were restricted from drinking alcohol the day preceding the study and participants were not allowed to have breakfast, coffee, or cigarettes before the experiment. Only water and tea with sugar was allowed. The participants either started the experiment at 9:00 or 11:00 in the morning. At arrival in the laboratory, the participant’s pupil size, HR, SBP, DBP, and subjective mood (indexed on a 9 by 9, pleasure by arousal grid; also see^44^) were assessed. Pupil measurements were repeated every twenty minutes before the visuomotor aiming task, again following immediately after the task, and again approximately thirty minutes later. HR, SBP, and DBP were assessed right before supplementation, and 60 and 90 minutes after supplementation (i.e., before and after the task). After the first assessment, participants ingested 400 ml orange juice with dissolved choline or placebo at arrival in the laboratory. Fourteen participants took choline in session one and placebo in session two, and the other fourteen participants vice versa. These sessions were separated by approximately 7 days (±1−3 days). The supplements were prepared in sealed containers by author MN and handed over to the naïve experimenter and none of the participants was able to determine a difference between the conditions.

Half an hour after supplementation, participants were provided a maximum of two apples or mandarins to prevent hunger while waiting for the behavioral task. The task was timed seventy minutes after intake to ensure that choline was taken up in the participant’s system. As such, participants waited more than an hour before conducting the behavioral task.

In each session, participants performed a spatial working memory and a visuomotor task which took approximately twenty minutes to complete. Here, we only present the results of the latter task because the results from the working memory task were inconclusive. The visuomotor task is almost identical to CANTAB’s Motor Screening Task (Cambridge Cognition Ltd, Cambridge, United Kingdom) in which participants have to point their finger at a target presented on a touch screen as fast and accurate as possible. In our task, however, participants had to use a computer mouse to move a cursor to a target as fast as possible and click as close to the target’s center as possible to gain points in each trial. A trial started with the presentation of a blank grey screen (Fig. 1b depicts a white background for aesthetical purposes), a fixation dot, and a mouse cursor for a duration randomly chosen from a uniform distribution within the range of 1 to 2 seconds (Fig. 1b). Next, the fixation dot disappeared and a circular target was shown at a random screen position. The target was 100 pixels in diameter (i.e., ~2.5 visual degrees) and consisted of a bulls-eye with 10 rings that alternated black and white as a function of eccentricity (the bulls-eye in Fig. 1b depicts less rings for aesthetical purposes). The target’s appearance released the mouse cursor and participants had to move the cursor to the target as fast as possible. Once the cursor arrived at the target, participants had to click the left mouse button with their right hand to hit the target as close to its center as possible. Participants could receive a maximum of 100 points when their reaction time was as fast as 250 ms (50 points) and accuracy right at the center (50 points). Reaction times slower than 250 ms decreased the score by 0.067 points per millisecond. A trial was automatically aborted when a participant did not hit the target within 1000 ms. Score also decreased as a function of hit distance to the target’s center (one point per pixel). A penalty of −100 points was given when target was missed or when the response was too late. After the response, participants received visual feedback for 1.5 s about their score in the current trial and their total score accumulated in previous trials. The task consisted of a total of 128 trials and each trial automatically started after the feedback.

### Analysis

Reaction times (RT) were based on the median time between target onset and target hit across all correct trials per participant (i.e., RT’s faster than one second and hits on the target). Hit distance was the mean Euclidian distance in visual degrees between the mouse cursor and target’s center at the time of the mouse click (i.e., the hit) across all correct trials. Number of misses was based on the amount of trials in which participant’s made an inaccurate movement and clicked the mouse while the cursor was off the target.

Pupil size was computed with custom software through several image processing steps. First, a manually set region of interest in the recorded images of the eye was transformed to binary image patches (i.e., two bit images with only zero or one values) by setting a luminance threshold per participant. Second, the center of the pupil was calculated by taking the median of *x* and *y* positions of all pixels in the darker region of the binary image (i.e., image locations with a zero value). Third, the pupil’s border was detected with a contrast analysis of 72 linear image lines (“rays”) that were extracted from the original image regions. The lines were radially aligned around the pupil’s center and extended from here to the periphery with a radius of 255 pixels (i.e., well beyond the pupil’s border). A strong increase in contrast in the image line indicated the pupil’s border. Last, pupil diameter was computed by taking the median of all radial distances from pupil center to the detected borders, resulting in an accurately fitted circle around the pupil (Fig. 1c). Due to strong camera zooming, which allowed accurate pupil size extraction, the depth of view got quite narrow and the proportional area of image covered by the pupil got large. These factors increased the likelihood that the pupil would move out of image borders or camera’s focus. Pupil size measurements therefore failed for two participants (both 18 years old; 1 female; body mass index 25.3 and 29.8; 1 took a placebo and the other choline in the first session) because they were unable to maintain their head fixed. For the same reason pupil measurements of two and three additional participants failed during the second-last and last recording, respectively.

We tested for the effects of choline on reaction times and hit distance with paired samples, two-sided t-tests. The percentage change in pupil size was calculated by subtracting and subsequently dividing pupil size in subsequent measurements by pupil size in the first measurement. Effects on pupil size were tested with a two-way repeated measure ANOVA with supplementation (choline vs. placebo) and measurement time after the first assessment at the start of the session as factors. Post-hoc one-tailed paired samples t-tests indicated at which time point choline significantly decreased pupil size as compared to placebo. We computed two-sided Pearson correlations coefficients to assess the relationship between changes in pupil size and differences in reaction time and accuracy between choline and placebo conditions across several time points during the experiment. Of particular interest was the pupil measurement right after the assessment of visuomotor performance because these correlations were subject to less noise due to the shorter time lapse between the measurements of the two variables. Missing data of the last pupil assessments of three participants were linearly interpolated for the ANOVA but not for the post-hoc comparisons and correlations. A false discovery rate correction was applied to the *p*-values of the correlations per assessment during the experiment^45^. We additionally conducted a bootstrap with replacement procedure on the correlation data to calculate the confidence intervals on 1000 newly acquired coefficients per data set.

## Results

**Figure 2:**
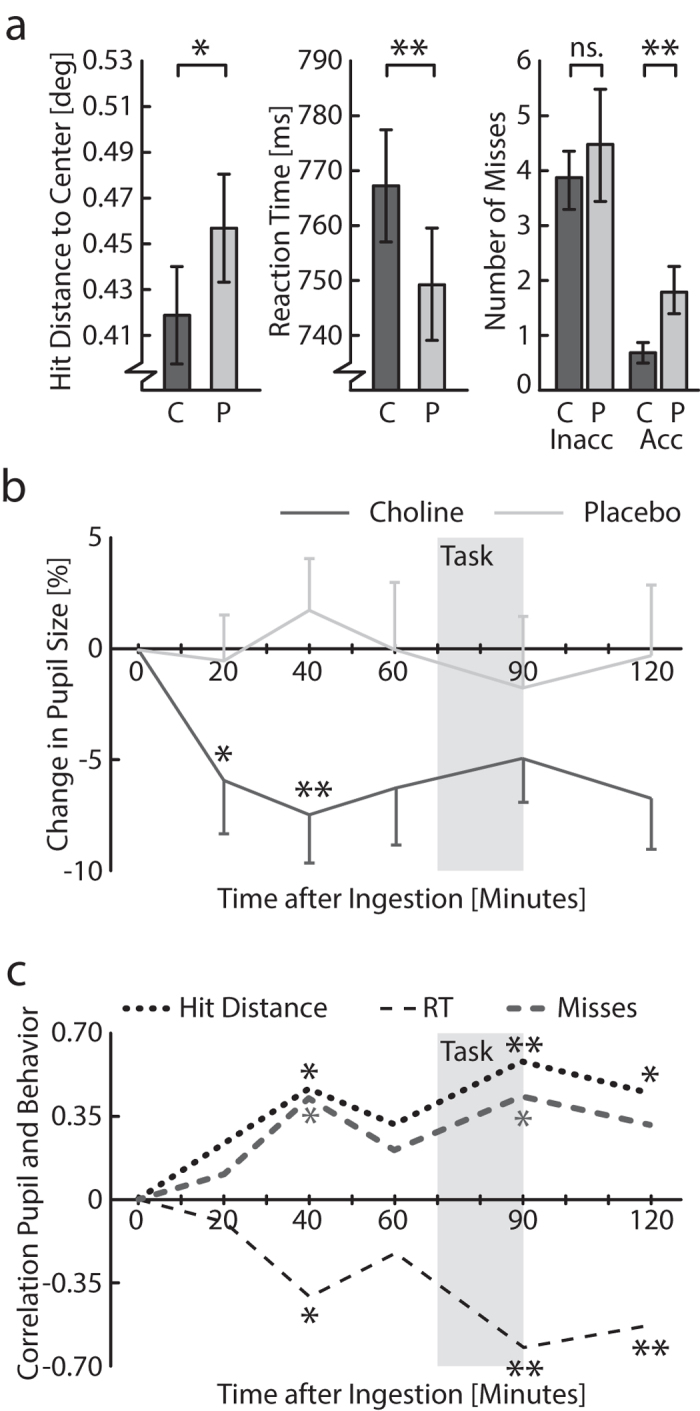
Results of the visuomotor performance task after choline and placebo supplementation. (**a**) As compared to the placebo condition (*light grey*), choline ingestion by the participants (*dark grey*) improved hit distance to the center of the target (*left*), slowed down reaction times (*center*), and decreased the amount of misses for participants that were most accurate (*right*) (C = choline; P = placebo; Inacc = inaccurate, participants with more misses; Acc = accurate, participants with less misses). (**b**) Participant’s pupil sizes also decreased as a function of time after choline (*dark grey*) but not placebo (*light grey*) supplementation. (**c**) Changes in pupil size correlated with hit distance to target center (*dotted, black*), reaction times (*dashed*), and number of misses (*dotted, grey*). Error bars represent the mean and standard error across participants (**a–b**) and asterisks (*p < 0.05, **p <0.01) indicate significance of t-test comparisons between choline and placebo conditions (a–b) or Spearman’s rho correlations between change in pupil size and task behavior (**c**). As indicated with the shaded area, the motoric aiming task was performed approximately 70–90 minutes after ingestion.

The results showed that targets were hit more towards the center after the ingestion of choline as compared to placebo (Fig. 2a; *t*(27) = 2.289, *p *= 0.030). Furthermore, we observed that reaction times to hit the targets were slower in the choline than placebo conditions (*t*(27) = 2.820, *p *= 0.009). The amount of misses showed a decreasing trend after choline administration (*t*(27) = 1.816, *p *= 0.080). A median group split analysis on this data showed that specifically participants with an overall small amount of misses were also more likely to decrease the amount of misses even more after choline ingestion (*t*(27) = 3.119, *p *= 0.008). Choline did not decrease the probability to miss for participants with an overall higher amount of misses (*t*(27) = 0.621, *p *= 0.546). Scores were also unaffected by choline supplementation (choline: M = 3750, SD = 794; placebo: M = 3813, SD = 793; *t*(27) = 0.450, *p *= 0.656). Taking all together, these results suggest that choline administration shifts the speed-accuracy trade-off of actions towards accuracy.

To test whether pupil size would reflect the impact of choline on the nervous system, we analyzed whether the pupil constricted after choline ingestion^38^. Indeed, pupil size after choline ingestion was significantly smaller than pupil size after a placebo (*F*(25,4) = 4.277, *p *= 0.0491). Post-hoc comparisons indicate that pupil size decreased significantly twenty and forty minutes after choline supplementation (Fig. 2b; *p’s *< 0.05). Furthermore, supplementation-induced changes in pupil size correlated positively with hit distance and number of misses, and negatively with reaction times (RT) at several time points (*p’s *< 0.05; for statistical details, see Supplementary Table S1), especially around the time that the task was performed (Fig. 2c; Hit distance: *r*(22) = 0.623, *p *= 0.001; RT: *r*(22) = − 0.579, *p *=  0.003; Misses: *r*(22) = 0.465, *p *= 0.022). These results indicate that a stronger decrease of pupil size was linked to slower reaction times and higher accuracy. In summary, choline supplementation biased the speed-accuracy trade-off towards more accurate responses, improving the spatial coordination of hand and arm movements.

## Discussion

We found that choline bitartrate administration improves visomotor performance and decreases pupil size of healthy humans. This is the first scientific evidence for a rapid change in the nervous system and behavior after choline ingestion, pointing at choline’s wide-ranging effects on cognition.

From learning to walk in early childhood to hitting a ball in sports, bodily movements are driven by a complex interplay of coordinated skeletal muscle movements. Muscles are innervated by networks of motor neurons that depend on neural communication through the chemical release of neurotransmitters. While the domain of psychopharmacology have extensively studied the effects of drugs on neurotransmitter release, blockage, or re-uptake inhibition (e.g.,^46^), few investigations have explored how nutrients are linked to nervous system activity and motor coordination. The present results demonstrate such a link and call for further research into the underlying mechanisms. For example, animal studies suggest that choline-to-acetylcholine synthesis acts centrally in the brain^11^,^12^,^47^. This chemical process may affect the cholinergic motor neurons that code and control the coordination of skeletal muscles (somatic nervous system) and pupillary muscles (autonomous nervous system). It has yet to be established precisely how this process functions in humans but recent indications suggest that cognitive control – a frontal lobe function^48^ – improves after CDP-choline supplementation^30^. Neighboring motor areas may also be affected by choline and recent evidence suggests that choline specifically targets nicotinic acetylcholine receptors^32^. Unfortunately, there are currently no non-invasive methods available to localize the actual chemical interactions of choline and acetylcholine in the human brain *in vivo*. However, follow-up studies could assess choline plasma levels in the participant’s blood to confirm the relation between choline uptake and behavioral changes because choline plasma levels increase after the intake of choline bitartrate or a similar substances^40^,^41^,^42^,^43^. Nonetheless, our findings point at the possibility that pupillometry could potentially be utilized as a tool to measure changes in nervous system activity after choline intake. Supported by a previous finding that showed decreases in heart rate and blood pressure after choline administration^49^, it is tempting to suggest that the choline administration increased parasympathetic nervous system activity. This would explain our observations of more focused, exploitative behavior, possibly due to altered cholinergic activity. While the noradrenergic sympathetic nervous system has received remarkably strong scientific interest, especially in the context of pupil dilations^50^ and the exploitation-exploration trade-off^51^,^52^, choline may counteract sympathetic activity through parasympathetic activity as reflected in the slower reaction times and decreases pupil size.

A particularly interesting observation from the present study was that individuals that were more effective (i.e., produced fewer misses) benefitted more from choline supplementation than less effective individuals. While this outcome pattern seems counterintuitive as one would expect more room for improvement in the less efficient individuals, we have observed similar patterns in other cognitive-enhancement interventions (e.g.,^53^). One possible explanation for such patterns might be that, at least in the cases being tested so far, the pre-interventional performance did not only reflect the current skill level but also the individual degree of short-term plasticity of the skill.

Previous studies reported no effect of choline supplementation on endurance in cyclists and runners^40^,^41^,^42^,^43^. In contrast to these studies, we have tested choline’s effects on visuomotor performance during rapid actions in response to visual displays in healthy students. Although we provided participants only one dose of choline, the results are in line with dietary (i.e., taking supplements for extended amount of times) animal studies that have demonstrated the important role of choline in the accurate development and maintenance of proper action coordination functions in rats^36^,^37^. The most obvious explanation for the null-results of previous studies on humans is that choline specifically affects fine motor control rather than ballistic movements and repetitive muscle movements as in cycling or running. However, we cannot exclude an interaction between choline and endurance exercise on cognition given the suggestion by Penry and Manore^54^ that exercise may increase brain choline concentration via synaptic plasticity and neurogenesis. Nevertheless, choline supplementation seems to have no effect on exercise performance among individuals with normal choline levels, but it has been observed among individuals with choline deficiency. Future studies need to clarify whether our observed results may be due to enhanced alpha-motor neuron function as well as improved neural signaling in the brain after choline ingestion.

The outcomes of this study may be of direct help to, for example, athletes who can benefit from improvements in motor coordination despite slower reaction times. The current sample consisted of healthy young students with well-developed cognitive performance. CDP-choline only seems to affect older populations or samples with relatively low cognitive performance, and it would be interesting to investigate whether choline bitartrate’s effects generalize to other populations and differs from CDP-choline’s specificity of action. As a final note to the general public, our findings together with previous findings^55^,^56^,^57^ suggest that eating foods rich in choline may provide several benefits, including improved motor coordination and health.

## Additional Information

**How to cite this article**: Naber, M. *et al*. Improved human visuomotor performance and pupil constriction after choline supplementation in a placebo-controlled double-blind study. *Sci. Rep*. **5**, 13188; doi: 10.1038/srep13188 (2015).

## Supplementary Material

Supplementary Information
