# Supplementary material for: Improved human visuomotor performance and pupil constriction after choline supplementation in a placebo-controlled double-blind study
Source: Sci Rep. 2015 Aug 14;5:13188. doi: 10.1038/srep13188 (PMC4536529; doi:10.1038/srep13188)
Supplement: Supplementary Information [file srep13188-s1.pdf]

Supplementary Information

Title: Improved human visuomotor performance and pupil constriction after choline supplementation in a placebo-controlled double-blind study

Authors: Marnix Naber, Bernhard Hommel, & Lorenza S. Colzato

Assess-ment	*n*	Behavioral Variable	Correlation Coefficient	Confidence Intervals	*p*-value	FDR corrected *p*
2 (20min)	26	Hit Distance	0.105	-0.2406 – 0.4708	0.6085	0.6426
		Reaction Time	0.237	-0.0125 – 0.5100	0.2507	0.6426
		Misses	-0.096	-0.3964 – 0.2010	0.6426	0.6426
3 (40min)	26	**Hit Distance**	**0.425**	**0.0286 – 0.7490**	**0.0306**	**0.0374**
		**Reaction Time**	**0.464**	**0.2121 – 0.6893**	**0.0170**	**0.0374**
		**Misses**	**-0.410**	**-0.7126 – -0.1057**	**0.0374**	**0.0374**
4 (60min)	26	Hit Distance	0.207	-0.1246 – 0.5507	0.3102	0.3102
		Reaction Time	0.314	0.0653 – 0.5531	0.1189	0.3102
		Misses	-0.225	-0.5160 – 0.0954	0.2690	0.3102
5 (90min)	24	**Hit Distance**	**0.465**	**0.1148 – 0.8636**	**0.0221**	**0.0221**
		**Reaction Time**	**0.579**	**0.3578 – 0.8426**	**0.0030**	**0.0046**
		**Misses**	**-0.623**	**-0.9244 – -0.2804**	**0.0011**	**0.0034**
6 (120min)	23	Hit Distance	0.312	-0.0660 – 0.7595	0.1480	0.1480
		Reaction Time	***0.445***	***0.1614*** **– 0.7777**	***0.0335***	*0.0502*
		**Misses**	**-0.528**	**-0.8608 – -0.1434**	**0.0096**	**0.0288**

Supplementary Table S1. Statistical details of correlations between changes in pupil size and visuomotor performance variables. Statistics of correlation coefficients between changes in pupil size (assessment 2-5) compared to baseline (assessment 1) and each behavioral variable from the visuomotor aiming task. Confidence intervals were computed with a bootstrap procedure with replacement. FDR means false-discovery-rate and significant correlations are printed in bold (or italic when FDR corrected was not significant). Correlation coefficients are also shown in Figure 2d.
